# Universal Access to Health and Universal Health Coverage: Nursing contributions

**DOI:** 10.1590/1518-8345.0000.2667

**Published:** 2016-03-28

**Authors:** Maria Helena Palucci Marziale

**Affiliations:** Chief Scientific Editor of the Revista Latino-Americana de Enfermagem and Full Professor of the Escola de Enfermagem de Ribeirão Preto, Universidade de São Paulo, PAHO/WHO Collaborating Centre for Nursing Research Development, Ribeirão Preto, SP, Brazil. E-mail: marziale@eerp.usp.br

**Figure:**
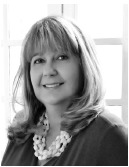

Universal Access to Health is considered to be the absence of sociocultural, organizational, economic, geographic and gender-related barriers in health care, and Universal Health Coverage to be the capacity of health systems to respond to the populations' needs at any care level, providing infrastructure, appropriately skilled human resources and health technologies without causing financial damage^(^ 1 ^)^. To respond to these demands, global actions are needed that involve different stakeholders.

What the activities of nursing human resources are concerned, the Resolution of the Pan American Health Organization (PAHO) "Human Resources for health: increasing access to qualified health workers in primary health care-based health systems"^(^ 2 ^)^ advocates the preparation of nurses with advanced practice qualifications to work in primary care services. Advance practice nurses have gained expert knowledge, complex decision making skills and clinical competences for advanced practice, whose characteristics are determined by the context and/or the country the worker is accredited to practice in. The entry level for this education is the Master's degree^(^ 3 ^)^.

In view of the inseparable relation among teaching, resarch and practice, nursing research priorities were listed for the region of the Americas, based on the concepts of Universal Access to Health and Universal Health Coverage, to guide nursing research on health systems and services. The six main categories in the priorities are: nursing human resource policies and education; structure, organization and dynamics of health systems and services; science, technology, innovation and information systems in public health; funding of health systems and services; health policies, governance and social control and social studies in health^(^ 4 ^)^. Further details on that list are available in the newly published articles by the Pan American Health Organization Regional Advisor on Nursing and Nursing Technicians and her collaborators in the American Journal of Nursing.

In support of international actions led by the Pan American Health Organization and the World Health Organization, to further the Universal Access to Health and the Universal Health Coverage, the Latin American Journal of Nursing, as the official scientific publication of the University of São Paulo at Ribeirão Preto College of Nursing and the PAHO/WHO Collaborating Centre for the Development of Nursing Research, has encouraged researchers, through calls for papers, to publish scientific contributions on the theme. In addition, the journal has invited some experts to present, based on background experiences, the contributions of Nursing to the strengthening of health systems in different countries, in view of the relevant role nursing professionals play in the delivery of health services.

In the set of articles published, the contributions resulting from the studies are highlighted, shown next.

"Health policies in conflict: insurance against universal public systems", which analyzed the results of the ongoing health reforms in Latin America, in terms of the guarantee of the right to health and the access to the services needed. Also, some strategies are proposed to strengthen the unified, public and solidary Health systems.

"Coverage, access and universal access in health: characteristics of scientific production in nursing", whose results indicate that, despite countless publications, research should be reinforced, constructed with the participation of the academy and community nursing.

"Education, leadership and partnerships: potentials of nursing for universal health coverage", in which the possibilities were discussed for nursing to contribute to universal health coverage, also presenting a call for nursing to encourage reflections and understanding about the relevance of its role on the route towards the consolidation of the principles of universal health coverage.

"The contribution of Portuguese nursing to universal access and coverage in health", analyzed through the identification of the nurses' distribution in the health system and the evolution of health indicators. The results indicate that nursing is the most numerous professional group in the Portuguese national health system, despite shortages in primary health care.

"Potential access to primary health care: what do the data from the program for better access and quality in Brazil show?", in which the influence of contextual indicators on cities' performance is analyzed, in the potential access to primary health care in Brazil. In addition, the contribution of nursing work to this access is discussed.

"Nurses' knowledge on universal health coverage for inclusive and sustainable elderly care services", developed based on the implementation strategies recommended by the WHO Global Forum for Governmental Chief Nursing Officers and Midwives, which reveals the existence of knowledge gaps among nurses in elderly care services. This requires attention in course curricula, also demanding the inclusion of public policy and advocacy themes.

These articles, as well as the other articles RLAE is publishing as from January 2016, are issued in the Rolling Pass modality. The adoption of this new publication format offers the possibility to shorten the time spent between the submission and the publication of articles, granting readers the opportunity to use the research results in their practice.

The total number of articles RLAE publishes per year does not change, nor do the journal volumes. The change refers to the elimination of the issue number. Hence, to cite the article, please follow the example below.
